# Thrombocytopenia Associated with Elemental Mercury Poisoning in Two Siblings — Connecticut, July 2022

**DOI:** 10.15585/mmwr.mm7238a2

**Published:** 2023-09-22

**Authors:** Emily W. Hogeland, Tarah S. Somers, Luke Yip, Suzanne Doyon, Carrie A. Redlich, Andrea D. Orsey, Craig B. Woda, Suzannah T. Swan, Henry M. Feder

**Affiliations:** ^1^Division of Pediatric Hospital Medicine, Connecticut Children’s, Hartford, Connecticut; ^2^Department of Pediatrics, University of Connecticut School of Medicine, Farmington, Connecticut; ^3^Agency for Toxic Substances and Disease Registry, Office of Community Health and Hazard Assessment, CDC; ^4^National Center for Environmental Health, Agency for Toxic Substances and Disease Registry, Office of Emergency Management, CDC; ^5^Connecticut Poison Control Center, Department of Emergency Medicine, University of Connecticut, Farmington, Connecticut; ^6^Department of Medicine, Occupational and Environmental Medicine Program, Yale University School of Medicine, New Haven, Connecticut; ^7^Division of Pediatric Hematology and Oncology, Connecticut Children’s, Hartford, Connecticut; ^8^Division of Pediatric Nephrology, Connecticut Children’s, Hartford, Connecticut; ^9^Pediatric Residency, University of Connecticut, Hartford, Connecticut; ^10^Division of Infectious Diseases and Immunology, Connecticut Children’s, Hartford, Connecticut; ^11^Department of Family Medicine, University of Connecticut School of Medicine, Farmington, Connecticut.

**Summary**

**What is already known about this topic?**

Elemental mercury vapor toxicity can manifest as a variety of clinical signs and symptoms, which can lead to delayed diagnosis, especially when exposure is not disclosed.

**What is added by this report?**

Two siblings, aged 5 and 15 years, experienced severe thrombocytopenia after elemental mercury vapor exposure. Infectious and hematologic etiologies were considered before the toxic exposure was recognized. Both children required chelation therapy, and the younger child had severe, protracted thrombocytopenia that required multiple medical interventions.

**What are the implications for public health practice?**

Elemental mercury vapor exposure is still a concern in residential settings, because mercury is used in the manufacture of fluorescent lighting and other devices, and it can still be found in glass thermometers and other sources. Prompt recognition of mercury toxicity and notification of public health authorities is essential for proper treatment and avoidance of further exposure.

## Abstract

Two siblings aged 5 and 15 years from Connecticut were hospitalized with petechial rash, oral mucositis, and severe thrombocytopenia approximately 10 days after they played with a jar of elemental mercury they found in their home. Before the mercury exposure was disclosed, the siblings were treated with platelet transfusions, intravenous immune globulin (IVIG) for possible immune thrombocytopenic purpura, and antibiotics for possible infectious causes. When their conditions did not improve after 6 days, poison control facilitated further questioning about toxic exposures including mercury, testing for mercury, and chelation with dimercaptosuccinic acid. The older sibling soon recovered, but the younger child required a prolonged hospitalization for severe thrombocytopenia, ultimately receiving repeated doses of IVIG, steroids, and romiplostim, a thrombopoietin receptor agonist. Close collaboration among multiple agencies was required to identify the extent of mercury contamination, evaluate and treat the other family members, and decontaminate the home. These cases demonstrate the importance of ongoing public health outreach to promote early detection of elemental mercury toxicity, and the need to evaluate for environmental exposures when multiple close contacts experience similar signs and symptoms.

## Clinical Presentations

Three weeks after moving into an older single-family home in Connecticut with her family, a girl aged 5 years (patient A) was evaluated at Connecticut Children’s emergency department with a 3-day history of petechial rash, oral ulcers, sore throat, chills, subjective fever, nose bleeds, and malaise. She appeared tired but stable, with vital signs notable for fever to 102.4°F (39.1°C), tachypnea (respiratory rate = 36 breaths per minute [normal for age = 18–25]), and tachycardia (pulse = 140 beats per minute [normal for age = 75–118]), but blood pressure measurement within normal limits for age and height (102/68 mmHg). She had tonsillar exudates and oral ulcerations, a petechial rash on her trunk and extremities, diffuse abdominal tenderness, and an enlarged spleen. The remainder of her physical examination findings were unremarkable, including respiratory examination with normal findings (apart from tachypnea). Results of laboratory tests were significant for severe thrombocytopenia (platelets <2,000/*μ*L [normal for age = 150,000–700,000]), anemia, eosinophilia, and elevated erythrocyte sedimentation rate and C-reactive protein, lactate dehydrogenase, and pro-brain natriuretic peptide, an indicator of poor cardiac function (Table). The patient’s chest radiograph indicated mild interstitial edema and borderline cardiac enlargement; subsequent echocardiogram results were normal except for mild tricuspid insufficiency. Abdominal ultrasound findings demonstrated splenomegaly.

**TABLE:** Laboratory values at hospital admission for two siblings with elemental mercury vapor poisoning — Connecticut, July 2022

Laboratory test, units	Laboratory results (hospital laboratory reference range)	
	Patient A (age 5 years)	Patient B (age 15 years)
WBC, thousand/*μ*L	5.8 (5.0–15.5)	5.6 (4.5–14.5)
WBC differential	42% neutrophils, 26% lymphocytes, 21% eosinophils, 9% monocytes	47% neutrophils, 34% lymphocytes, 4% eosinophils, 15% monocytes
Hgb, g/dL	9.9 (10.6–14.6)	13.1 (11.4–15.4)
Hematocrit, %	30.6 (32–43.8)	40.1 (34.2–46.2)
Platelets, thousand/*μ*L	<2 (150–700)	<2 (150–450)
Blood urea nitrogen, mg/dL	8 (5–18)	15 (5–18)
Cr, serum, mg/dL	0.4 (0.2–0.7)	0.7 (0.5–1.3)
Aspartate aminotransferase, U/L	30 (9–45)	98 (10–55)
Alanine transaminase, U/L	7 (10–50)	99 (10–55)
Albumin, g/dL	3.4 (3.8–5.4), stable	3.2 (3.2–4.5), 2 days later, declined to 1.9
Lactate dehydrogenase, U/L	483 (120–300)	581 (120–300)
CRP, mg/dL	5.1 (<0.5)	2 (<0.5)
ESR, mm/hr	43 (3–13)	95 (<15)
Ferritin, *μ*g/L	327 (15–150)	—
Pro-brain natriuretic peptide, pg/mL	961 (<125)	—
Urinalysis	Tr Hgb; Tr leukocytes; negative protein; microscopy not performed	Lg Hgb; Lg protein; 2 RBC, up to >25 by hospital day 6, urine protein/Cr ratio >2.9
Mercury concentration, whole blood, *μ*g/L*	315 (<10)	518 (<10)
Mercury concentration, random spot urine, *μ*g /gCr^†^	409 (<4)	>1,000 (<4)

**Abbreviations:** Cr = creatinine; CRP = C-reactive protein; ESR = erythrocyte sedimentation rate; Hgb = hemoglobin; Lg = large; NHANES = National Health and Nutrition Examination Survey; RBC = red blood cells; Tr = trace; U = units; WBC = white blood cells.

* Whole blood: reference range of <10 *μ*g/L is from Quest Diagnostics. 2017–2018 NHANES 95th percentiles are 0.960 *μ*g/L for age 1–5 years (patient A) and 1.71 *μ*g/L for age 12–19 years (patient B). https://www.cdc.gov/exposurereport/data_tables.html

^†^ Random spot urine: reference range of <4 *μ*g/gCr is from Quest Diagnostics. 2017–2018 NHANES 95th percentiles are 0.923 *μ*g/gCr for age 3–5 years (patient A) and 0.643 *μ*g/gCr for age 12–19 years (patient B). https://www.cdc.gov/exposurereport/data_tables.html

The patient’s signs and symptoms, the abnormal laboratory findings, and the recognized potential for exposure to multiple pets and mice in the home, led to consideration of a broad range of bacterial (including tickborne) and viral diseases, as well as immune thrombocytopenic purpura, an immune-mediated clotting disorder. The child was treated empirically with ceftriaxone and doxycycline for coverage of tickborne diseases. Her fever resolved within 24 hours; however, severe thrombocytopenia (platelets <10,000/*μ*L) persisted despite a platelet transfusion and administration of intravenous immune globulin (IVIG). On the sixth hospital day, she underwent bone marrow aspiration and biopsy that revealed normocellular marrow with normal trilineage hematopoiesis (i.e., the production of platelets, red blood cells, and white blood cells that is not indicative of malignancy or bone marrow failure), and hematopoietic cellularity of 80% with megakaryocytic hyperplasia demonstrating normal bone marrow function with increased platelet precursor cells. In addition, small nonnecrotizing granulomas, a nonspecific indicator of inflammation, were present.

At the time of patient A’s evaluation, her maternal half-brother aged 15 years (patient B) was also evaluated at the same hospital for similar complaints, including a 4-day history of petechial rash, oral ulcers, chills, fatigue, and abdominal pain. When examined, he appeared anxious, but he was afebrile; his blood pressure measurement was elevated (142/63 mm Hg [normal for age <120/80]), he was tachypneic (respiratory rate = 27 [normal for age = 12–20]) and mildly tachycardic (pulse = 106 [normal for age = 60–100]). Physical examination revealed a petechial rash on his trunk and extremities, mouth ulcers, conjunctival injection, and abdominal pain. Apart from his tachypnea, findings from examination of his respiratory system were normal. Although edema was not observed initially, mild bilateral lower extremity edema was subsequently observed. Laboratory findings were notable for severe thrombocytopenia (platelets <2,000/*μ*L [normal for age = 150,000–450,000]), elevated liver enzymes (aspartate aminotransferase and alanine transaminase), elevated erythrocyte sedimentation rate and C-reactive protein, hypoalbuminemia, nephrotic-range proteinuria, and microscopic hematuria. Similar to those of patient A, findings from patient B’s abdominal ultrasound demonstrated splenomegaly. His chest radiograph was normal. He also received empiric ceftriaxone and doxycycline. Given the findings of elevated blood pressure measurements and a serum albumin level of <2.5g/dL, his treatment included fluid restriction and a low sodium diet to reduce the overall risks for worsening of nephrotic syndrome (clinically diagnosed based on findings of nephrotic-range proteinuria, low albumin, and edema). Thrombocytopenia (≤3,000/*μ*L) persisted despite receipt of two platelet transfusions and IVIG.

## Investigation and Outcomes

### Toxic Exposure Evaluation

Because results of the patients’ infectious disease evaluations remained inconclusive after 6 days of hospitalization, the possibility of a toxic exposure causing the observed signs and symptoms was considered, and the Connecticut Poison Control Center (CPCC) was contacted. Medical toxicologists suggested the possibility of elemental mercury exposure and asked that further history be obtained. Patient B then disclosed that patient A had found a small jar (6–8 fluid ounce capacity) full of mercury approximately 10 days before their hospital admission. The mercury spilled on the carpeted floor of a second-floor bedroom. The boy spent about 30 minutes attempting to clean up the spill with his hands, and he estimated that he was able to collect approximately one half of the mercury and replace it in the jar. Other family members were unaware of the spill. The boy continued to sleep in the room, and the girl continued to play there. A week after the spill (several days before admission), another family member used a vacuum cleaner on the carpet in the room. Whole blood mercury concentrations for patients A and B were 315 *μ*g/L and 518 *μ*g/L, respectively, compared with the normal range of <10 *μ*g/L.

### Environmental Surveillance and Identification of Additional Cases

At the recommendation of the CPCC, the Connecticut Department of Energy and Environmental Protection (DEEP) was immediately contacted. In collaboration with the local health department, the family was evacuated from the home without delay and placed in temporary housing. Using mercury vapor analyzers recommended for the site conditions, concentrations of mercury vapor in ambient air were 174.6 *μ*g/m^3^ outside the closed front door, 99.4 *μ*g/m^3^ outside the closed back door, and >999 *μ*g/m^3^ on the second floor (the location of the spill), saturating the sensor. During a mercury spill response, the Agency for Toxic Substances Disease Registry (ATSDR) and the Environmental Protection Agency have recommended that indoor air mercury vapor concentrations of ≥1 *μ*g/m^3^ are unacceptable for normal residential occupancy (*1*).

The seven other family members living in the home received testing for mercury poisoning and results indicated elevated whole blood mercury concentrations (range = 125–310 *μ*g/L [normal <10 *μ*g/L]) for all of them; they were all then referred to an environmental medicine clinic for further evaluation. As part of the ongoing response, extensive efforts were undertaken to characterize the mercury contamination within the home. Results indicated that exposures to the family members were highest in the bedroom where the spill occurred and where the children spent time. The jar of mercury was in the house before the family obtained the title to the property, and its original purpose is unknown. The home was successfully decontaminated, and the family was eventually able to return.

### Hospital Treatment and Outcomes

Upon disclosure of the mercury exposure, both siblings were treated with dimercaptosuccinic acid* for chelation (used off-label; non-Food and Drug Administration (FDA)–approved indication). Patient B was discharged on hospital day 15 with a platelet count of 47,000/*μ*L and subsequently completed a 19-day course of chelation as an outpatient (Figure). Although dipstick urinalysis results continued to show proteinuria and hematuria, levels of his urine protein-to-creatinine ratio normalized at 0.1 mg per mg. Aided by fluid and salt restriction, the patient’s blood pressure measurements normalized within 2–3 days of starting chelation.

**FIGURE:**
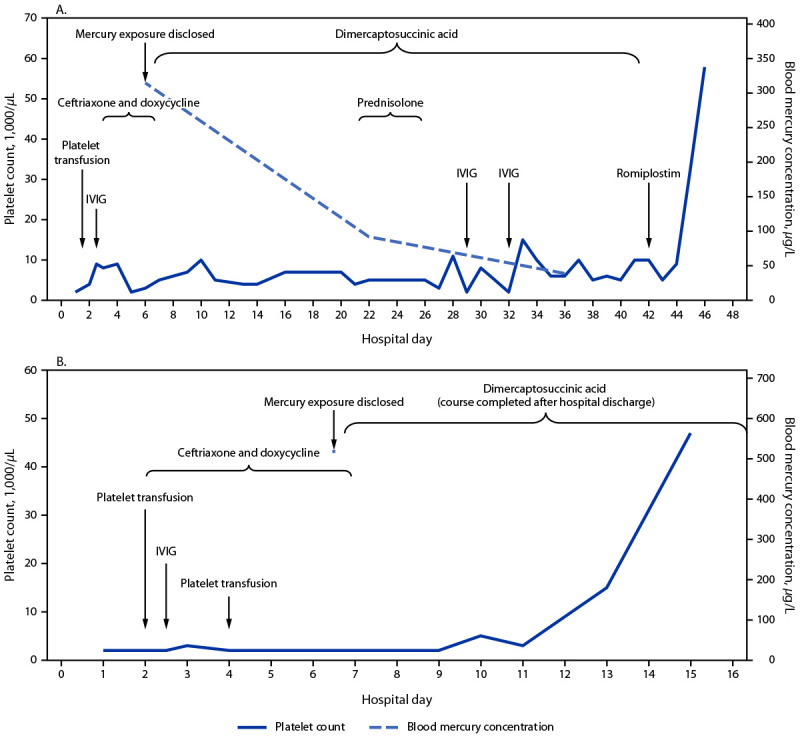
Platelet count,* blood mercury concentration,^†^ and interventions^§^ for two siblings,^¶^ patient A, aged 5 years (A) and patient B, aged 15 years (B) with elemental mercury vapor poisoning resulting from an exposure in the home — Connecticut, July 2022 **Abbreviation: **IVIG = intravenous immune globulin. * Platelet counts were <2,000 per/*μ*L on hospital day 1 (patient A) and hospital days 1, 2, 4, 5, 6, 7, 8, and 9 (patient B). ^†^ Blood mercury concentration was measured on only one occasion (hospital day 6) for patient B during hospitalization. ^§^ Dimercaptosuccinic acid is a chelating agent; romiplostim is a bone marrow stimulant. ^¶^ The x-axis ranges in panels A and B are different. Patient A was discharged on hospital day 46, and patient B was discharged on hospital day 15.

In contrast, patient A continued to experience thrombocytopenia, despite an extended chelation course with dimercaptosuccinic acid (19-day initial course followed by a subsequent 16-day course), initial platelet transfusion, a steroid pulse, and a total of three IVIG infusions. On hospital day 42, treatment with weekly romiplostim,^†^ a thrombopoietin receptor agonist (used off-label; non-FDA–approved indication) was initiated, resulting in rising platelet count, and she was discharged on hospital day 46 with a platelet count of 58,000/*μ*L and a whole blood mercury concentration of 39 *μ*g/L. She continued to receive romiplostim therapy for a total of 7 weekly doses until results of her platelet count normalized at 250,000/*μ*L; and the platelet count remained normal after discontinuation of romiplostim.

## Discussion

Elemental mercury poisoning is rare and can manifest as various central nervous system, liver, kidney, hematologic, skin, and cardiovascular abnormalities, which can lead to delays in diagnosis (*2*). CDC and ATSDR warn that inhalation of elemental mercury vapors is a known health hazard.^§^ Because of the volatility of mercury at room temperature, even small spills can generate dangerous mercury vapor concentrations indoors. Using a vacuum cleaner on a spill can increase vaporization of mercury and spread it to different parts of a home. Children are at higher risk for harmful exposures because of their physiology and closer proximity to vapors from mercury spills on the ground.^¶^ Although mercury-containing devices are becoming less common in the home, sources of elemental mercury vapor (such as broken compact fluorescent light bulbs and glass thermometers) still exist and mercury spills in residential buildings remain a concern.

Hematologic effects from elemental mercury vapor poisoning affecting all cell lines have been reported but remain rare (*3*). Although autoimmune thrombocytopenia and fevers have also been reported from mercury vapor poisoning (*4*,*5*), these two cases of severe thrombocytopenia were likely due to high blood mercury concentrations resulting from approximately 2 weeks of exposure, with patient A’s illness representing a particularly protracted case. Test results for Patient A’s bone marrow displayed megakaryocytic hyperplasia, suggesting that the thrombocytopenia she experienced was likely due to peripheral platelet destruction, possibly immune-mediated, which led to splenomegaly from sequestration. In addition to thrombocytopenia, evidence of nephrotic syndrome was observed for patient B and was likely related to membranous nephropathy from mercury toxicity, which has been previously reported (*6*).

### Public Health Implications

These cases highlight the importance of ongoing public health education and outreach to facilitate early detection of elemental mercury toxicity, and the need to consider environmental exposures (in addition to infectious etiologies) when multiple household members experience similar signs and symptoms. ATSDR has prepared materials describing the health effects of mercury exposure, and instructions for proper mercury disposal.** Timely notification of local public health and environmental protection agencies is critical for the safe cleanup of elemental mercury spills and possible evacuation or relocation of persons whose homes are found to have unsafe levels of mercury. 

This activity was reviewed by CDC, deemed not research, and was conducted consistent with applicable federal law and CDC policy.^††^

## Footnotes

*
https://www.accessdata.fda.gov/drugsatfda_docs/label/2018/019998s021lbl.pdf

†
https://www.accessdata.fda.gov/drugsatfda_docs/label/2021/125268s167lbl.pdf

§
https://www.atsdr.cdc.gov/toxfaqs/tfacts46_metallic_mercury.pdf

¶
https://www.atsdr.cdc.gov/mercury/docs/mercuryrtcfinal2013345.pdf

**
https://www.atsdr.cdc.gov/mercury/index.html

†† 45 C.F.R. part 46, 21 C.F.R. part 56; 42 U.S.C. Sect. 241(d); 5 U.S.C. Sect. 552a; 44 U.S.C. Sect. 3501 et seq.
